# Beyond output metrics: reframing AI-assisted vocal pedagogy through human learning and educational value

**DOI:** 10.3389/fpsyg.2026.1844841

**Published:** 2026-07-01

**Authors:** Youhe Li

**Affiliations:** Shenzhen Technology University, Shenzhen, China

**Keywords:** AI-assisted feedback, artificial intelligence, educational value, embodied cognition, metacognition, music education, self-regulated learning, vocal pedagogy

## Abstract

Artificial intelligence is increasingly entering music and performance education, often through tools that measure pitch, stability, timing, and related performance indicators. In vocal pedagogy, however, more precise measurement does not automatically create educational value. This Perspective argues that AI-assisted vocal pedagogy should be evaluated by how AI-generated evidence becomes meaningful for human learning: how learners interpret feedback, regulate practice, sustain motivation, and develop trust in teacher-guided learning processes. The conceptual problem is that measurable vocal outputs may not fully represent learners’ internal experience, embodied coordination, or expert pedagogical interpretation. Drawing on educational psychology, performance science, and recent work on AI-supported and metacognitive music learning, the article proposes a framework that links technical adaptation, human learning processes, and educational outcomes. Within this framework, effectiveness, equity, and sustainability are treated as outcome criteria for judging whether AI-supported feedback becomes meaningful for learning, usable across learners, and sustainable for long-term vocal development. The article concludes that AI should not be positioned as an autonomous evaluator of singing quality, but as a human-centered support for interpretation, reflection, teacher–student dialogue, and pedagogically responsible decision-making.

## 1 Introduction

Vocal pedagogy has long been grounded in forms of knowledge that are difficult to reduce to external measurement. Singing teachers listen, demonstrate, use sensory and metaphorical language, and make pedagogical judgments in relation to the learner’s body, repertoire, genre, vocal condition, and developmental trajectory. This tradition remains central because singing is a bodily, interpretive, expressive, and developmental form of learning, not merely a measurable acoustic output to be corrected (Sundberg, 1987; Titze, 2000; Crocco et al., 2020). At the same time, this individualized tradition can make feedback difficult to standardize, limit access to process-based evidence, and leave learners unsure how to translate evaluative comments into concrete practice strategies.

AI enters vocal pedagogy at a transitional moment. In music and performance education, AI-assisted and technology-enhanced tools are increasingly used to analyze, visualize, and organize measurable aspects of performance (Waddell et al., 2019; Macrides and Angeli, 2020; Li et al., 2025). Recent singing voice research also shows that vocal learning and performance involve motor-learning, longer-term vocal development, and health-related considerations that require pedagogically sensitive interpretation (Crocco et al., 2020; Nusseck et al., 2021; Zuim et al., 2024). These developments create genuine educational opportunities by making some aspects of vocal performance more visible and available for reflection and teaching.

The same transition also creates a pedagogical risk. If AI-generated evidence is treated as educational value in itself, vocal training may be narrowed into output correction, score optimization, or automated judgment. A pitch curve, stability score, vibrato display, or other performance indicator may appear precise, but it does not by itself explain what the learner experienced, why a pattern occurred, or what instructional response would support development. This concern is consistent with broader educational guidance that AI should support human-centered and educationally meaningful learning processes (UNESCO, 2023; OECD, 2023).

This Perspective examines how AI-generated vocal evidence can become educationally meaningful. It proposes a framework linking three levels: technical adaptation, human learning processes, and educational outcomes. The framework begins with the evidence AI makes visible, examines how that evidence is interpreted through bodily experience, cognition and metacognitive monitoring, self-regulated practice, motivation, learner beliefs, and pedagogical mediation, and then asks whether this process produces educational value.

Existing AI in education discussions often foreground technological capability, responsible use, equity, and policy guidance. Feedback literacy frameworks explain how learners understand and act on feedback, and self-regulated learning frameworks explain planning, monitoring, and strategy adjustment. These perspectives are important, but they do not fully explain how measurable vocal evidence becomes pedagogically usable in voice teaching, where genre, embodied sensation, teacher judgment, learner readiness, and developmental goals shape what evidence means. This Perspective brings these strands together in a voice-specific framework: AI-supported tools make measurable performance features visible, teachers and learners interpret them through human learning processes, and educational outcomes indicate whether the evidence supports vocal development over time.

### 1.1 Scope of this perspective

This Perspective adopts a problem-driven conceptual approach. Its central argument is that AI-assisted vocal pedagogy should be evaluated not only in terms of output accuracy or technical optimization, but also in relation to human learning, interpretation, teacher–student interaction, and educational value.

The discussion draws on five bodies of literature: singing voice science, vocal pedagogy, embodied music cognition, and technology-enhanced music learning; feedback, formative assessment, self-regulated learning, and feedback literacy; metacognition, reflective practice, and self-regulated music practice; recent AI-assisted vocal and music learning; and human-centered and responsible AI in education. These sources are used to connect performance evidence, feedback interpretation, learner regulation, pedagogical mediation, and educational responsibility.

Foundational and field-building literature from the late twentieth century to around 2020 provides the conceptual basis for singing voice production, embodied performance, feedback, metacognition, self-regulated learning, and feedback literacy. Recent literature, especially from approximately 2021 to 2026, is used where the manuscript addresses AI-supported learning, music education, performing arts pedagogy, learner beliefs, learner agency, and responsible educational technology. Together, these bodies of literature support a framework that links technical adaptation, human learning processes, and educational outcomes in AI-assisted vocal pedagogy. Effectiveness, equity, and sustainability are treated as outcome criteria for judging whether AI-supported feedback becomes meaningful for learning, usable across learners, and sustainable for long-term vocal development.

## 2 From technical adaptation to the problem of educational value

Technical adaptation marks the first level of AI-assisted vocal pedagogy. In music and performance education, digital and AI-supported tools have been used to support assessment, feedback, revision, and performance monitoring (Waddell et al., 2019; Macrides and Angeli, 2020). Multimodal approaches have expanded the kinds of evidence available for understanding performance states, including physiological or state-related information alongside musical features (Horwitz et al., 2021; Soliński et al., 2024). Recent work has also examined AI-assisted feedback and AI-supported practice in music and vocal training contexts (Li et al., 2025; Ou et al., 2025).

The educational problem begins when technical evidence is expected to become meaningful for learning. AI-generated output becomes valuable only when it is interpreted, taken up, and used within teaching and practice. Feedback becomes pedagogically meaningful when learners and teachers connect it with prior experience, use it to support monitoring and strategy adjustment, and incorporate it into self-regulated practice (Nicol and Macfarlane-Dick, 2006; Hattie and Timperley, 2007; Carless and Boud, 2018; Li et al., 2025; López-Íñiguez and McPherson, 2024). In vocal pedagogy, AI-generated metrics must therefore be translated into bodily awareness, technical coordination, expressive decision-making, and pedagogical dialogue.

This problem is especially clear in singing because similar observable outcomes may carry different pedagogical meanings. Similar acoustic stability, pitch accuracy, or vocal performance indicators may reflect different forms of bodily coordination and sensorimotor engagement (Leman, 2007; Bremmer and Nijs, 2020). A more stable output may indicate more efficient coordination, but it may also accompany overcontrol, reduced expressive exploration, or increased dependence on external correction. Temporary instability may reflect productive experimentation or a learner’s attempt to integrate new forms of control. The educational significance of AI-generated evidence therefore depends on how measurable output is connected with effort, perception, control, confidence, expressive intention, and developmental readiness.

**Figure 1:**
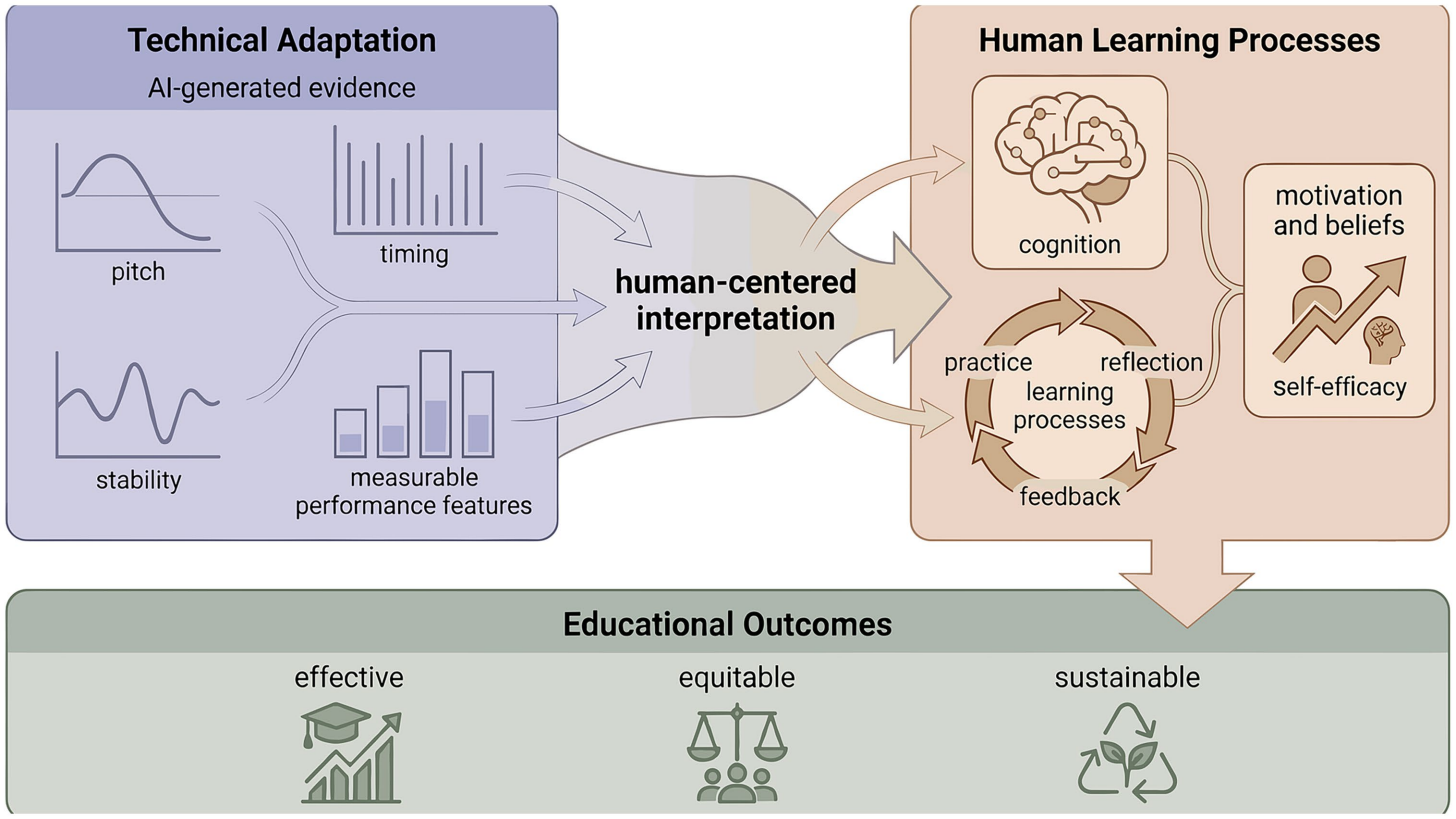
Conceptual framework for interpreting AI-assisted vocal pedagogy beyond technical adaptation. This figure presents an interactive framework linking technical adaptation, human learning processes, and educational outcomes. The first layer represents AI-generated evidence, including acoustic, performance-related, and multimodal indicators. The second layer represents the interpretive and pedagogical processes through which this evidence is connected with bodily experience, cognition and metacognitive monitoring, self-regulated practice, motivation, learner beliefs, and teacher–student dialogue. The third layer represents educational outcomes, where effectiveness, equity, and sustainability function as outcome criteria for judging whether AI-supported evidence has become meaningful for learning. The arrows should be read recursively rather than linearly: evidence informs interpretation, interpretation shapes practice, practice changes later evidence, and later evidence invites further pedagogical response.

The framework follows from this gap between technical evidence and educational meaning. It asks three linked questions: what evidence does AI make visible, how is that evidence interpreted through human learning processes, and what educational outcomes follow? Figure 1 visualizes this as a dynamic relationship among three levels. Measurable performance features, such as pitch deviation, acoustic stability, vibrato-related patterns, or timing regularity, do not carry a single pedagogical meaning. Their pedagogical interpretation varies according to genre, task purpose, stylistic expectation, bodily sensation, learner readiness, and teacher judgment. The same measured deviation may indicate technical inaccuracy, expressive inflection, or a recording/context issue. Interpretation then guides feedback, practice, and reflection. Later practice produces new vocal evidence, so the model should be read as recursive rather than as a one-way pipeline from measurement to outcome.

## 3 Human learning processes as the mediating layer of the framework

Human learning processes form the mediating layer between technical adaptation and educational outcomes. AI feedback enters learning through interpretation: it must be connected with bodily experience, evaluated against prior knowledge, and transformed into practice decisions. Four educational-psychological mechanisms are especially important: metacognitive interpretation and monitoring, self-regulated practice and feedback uptake, motivational and affective conditions, and learner beliefs, agency, and dependence.

First, AI feedback becomes meaningful only when learners can interpret it. In vocal training, learners must relate external evidence to bodily sensation, perceived effort, vocal control, and future adjustment. This makes metacognition central to the educational use of AI feedback (Flavell, 1979; Araújo et al., 2024). Research on music learning links metacognition with planning, monitoring, evaluation, and practice regulation (Concina, 2019), while recent work emphasizes bodily awareness, reflective evaluation, and strategic adjustment in musical practice (Araújo et al., 2024; Zhang, 2025; López-Íñiguez and McPherson, 2024).

Second, AI-generated evidence must enter self-regulated practice rather than remain a detached correction. Singing is a dynamic and embodied form of skill acquisition involving cycles of action, sensation, feedback, adjustment, and renewed practice (Leman, 2007; Bremmer and Nijs, 2020), and music learners need to monitor and regulate practice over time (McPherson et al., 2018). AI feedback can support this process when it helps learners notice patterns, set goals, select strategies, and reflect. It may increase dependence when it substitutes for internal monitoring, but it can strengthen autonomous regulation when it supports self-monitoring (McPherson et al., 2019; Peng et al., 2026). Recent higher-music-education research similarly emphasizes reflection, adjustment, and learner uptake in formative assessment and self-assessment (Zhang et al., 2026).

Third, motivation, affect, and learner beliefs shape how AI evidence is accepted, resisted, or used. This is especially important in singing, where feedback is tied to self-expression, vulnerability, and vocal identity. A technically correct correction may support development when it is experienced as usable guidance, but it may disrupt learning when it is experienced as fixed judgment, excessive error exposure, or loss of control (Efklides, 2011; Ouyang, 2025). Students also differ in how they understand ability, effort, progress, and the authority of external systems. Recent music teacher education research similarly suggests that performance expectancy and effort expectancy shape intentions to use AI in music education contexts (Niu, 2026). Human learning therefore remains interpretive, situated, and belief-laden (Efklides, 2011; Peng et al., 2026).

These mechanisms show why technical evidence cannot be evaluated apart from human learning. Between measurement and educational value lies an interpretive learning process in which learners understand evidence, regulate practice, sustain motivation, and position themselves in relation to AI, teachers, and their own developing voice.

## 4 Operationalizing educational value in the proposed framework

Educational value can be examined by distinguishing technical indicators, interpretive standards, learning processes, and educational outcomes. A technical indicator is what AI makes visible; an interpretive standard asks what that indicator means in context; a learning process concerns how learners and teachers use the evidence; and an educational outcome concerns the development that the process supports. Educational value is therefore not demonstrated by separating technical indicators from educational indicators. The same evidence may remain merely technical if it is treated as a score, but may become educationally meaningful when it supports interpretation, action, uptake, mediation, or long-term development.

### 4.1 Technical indicators and interpretive standards

AI systems may identify pitch deviation, timing, acoustic stability, vibrato-related features, intensity, practice traces, or other performance-related evidence. These indicators can be useful, but they do not have fixed pedagogical meanings in isolation. A pitch deviation may reflect listening, registration, fatigue, task difficulty, expressive pressure, or stylistic choice. Measurement reliability concerns whether evidence is consistent across tasks, devices, and recording contexts; measurement validity concerns whether its interpretation is appropriate for a specific pedagogical decision.

### 4.2 Actionability, uptake, and pedagogical mediation

Educational value also depends on whether AI-generated evidence can be acted upon. Feedback becomes actionable when it helps learners decide what to attend to, what to try, and when to seek teacher explanation (Nicol and Macfarlane-Dick, 2006; Hattie and Timperley, 2007; Carless and Boud, 2018). Uptake appears when learners develop clearer practice goals, better effort monitoring, or greater ability to discuss vocal problems with the teacher (López-Íñiguez and McPherson, 2024; Zhang et al., 2026). Teachers mediate this process by explaining feedback, filtering irrelevant indicators, contextualizing genre and task demands, and helping learners decide whether a measured pattern should be corrected, explored, or temporarily accepted as part of development.

This mediation is also where equity enters the framework. Feedback that is clear to an advanced learner may be confusing to a novice; feedback that assumes technical vocabulary, bodily awareness, or stable confidence may not function equally well across learners. AI-assisted feedback becomes equitable when it is made usable across differences in training background, language resources, bodily awareness, confidence, and interpretive readiness (de Bruin, 2024; OECD, 2023; Garcia Ramos and Wilson-Kennedy, 2024; Varsik and Vosberg, 2024).

### 4.3 Educational value outcomes

Effectiveness, equity, and sustainability function here as outcome criteria for judging whether AI-generated evidence has become meaningful in practice (OECD, 2023; UNESCO, 2023). Effectiveness concerns whether feedback strengthens understanding, strategy adjustment, self-monitoring, and reflective practice rather than only short-term accuracy (Hattie and Timperley, 2007; Li et al., 2025; McPherson et al., 2019). Equity concerns whether evidence can be interpreted and used across learner differences (OECD, 2023; Garcia Ramos and Wilson-Kennedy, 2024; Varsik and Vosberg, 2024). Sustainability concerns whether AI support remains beneficial over time by strengthening self-regulation, expressive development, teacher judgment, and learner autonomy while preserving the temporal realities of human skill development (UNESCO, 2023; Peng et al., 2026).

Table 1 complements Figure 1 by translating the framework into operational criteria and possible empirical uses.

**Table 1:** From technical evidence to educational value: operational criteria for AI-assisted vocal pedagogy.

Framework question	Operational focus	Evidence and interpretive standard	Educational value addressed	Possible empirical use
What evidence does AI make visible?	Measurement validity and reliability	Evidence: pitch deviation, timing, vibrato-related patterns, intensity, practice traces. Standard: reliable across tasks and recording conditions; valid for the teaching purpose.	Basis for effective feedback: measurement and interpretation fit the pedagogical goal.	Test–retest checks; recording-context comparison; error analysis; expert–AI agreement.
How is evidence interpreted in context?	Context-sensitive interpretation	Evidence: scores, labels, displays, feedback statements. Standard: read against task, genre, effort, coordination, and expressive intention.	Pedagogical sense-making: meaning and next pedagogical action are explainable.	Explanation tasks; interviews; response-process evidence; AI–expert–learner alignment.
How does evidence support learning?	Feedback actionability and uptake	Evidence: prompts, practice suggestions, comparisons, reflective cues. Standard: identifies a practice focus, strategy, or next step.	Strategy adjustment: feedback changes practice quality, not only awareness.	Practice plans/logs; stimulated recall; learner uptake; strategy-adjustment analysis.
How can evidence become usable across learners?	Pedagogical mediation and equitable access	Evidence: teacher–student dialogue, contextualized evidence, adapted feedback, filtered indicators. Standard: usable across learner readiness, vocabulary, confidence, and bodily awareness.	Equitable access: different learners can use feedback with appropriate mediation.	Learner-profile comparison; usability/comprehension tasks; AI-only vs. teacher-mediated conditions.
What educational development follows over time?	Developmental sustainability and transfer	Evidence: feedback history, learner reflection, delayed performance, transfer beyond the tool. Standard: strengthens autonomy rather than dependence.	Sustainable development: self-regulation, expressive development, transfer, and evaluative judgment over time.	Longitudinal/repeated-measures studies of self-regulation, trust, dependence, and transfer.
What conditions shape responsible assessment?	Responsible assessment context	Evidence: model assumptions, training data, transparency, privacy, fairness, and recording/task/genre context. Standard: reviewed before evaluative use.	Human-centered safeguards: fairness, privacy, transparency, and context sensitivity.	Bias/fairness audit; privacy review; transparency notes; robustness testing.

## 5 Discussion

The framework developed in this Perspective reframes AI-assisted vocal pedagogy as a problem of educational interpretation rather than technical adaptation alone. AI can make vocal evidence more visible, but visibility does not by itself create educational value. Pitch accuracy, vibrato-related patterns, acoustic stability, effort-related signals, and AI-generated feedback statements remain educationally incomplete unless they are interpreted through human learning processes, pedagogically mediated, and used to support practice.

This argument clarifies how Figure 1 should be read. The model is interactive and recursive rather than a simple linear pipeline from measurement to outcome: AI-generated evidence informs interpretation; interpretation shapes pedagogical and practice decisions; practice changes later vocal evidence; and later evidence invites further response. The framework explains the movement from evidence visibility to educational value, with effectiveness, equity, and sustainability as evaluative outcomes. Table 1 then translates this movement into operational criteria and possible empirical uses.

For singing voice assessment, AI-assisted systems can organize acoustic evidence, feedback, and reflective comparison, but vocal quality and development cannot be reduced to output metrics alone. Measurable results must be interpreted in relation to effort, coordination, expressive intention, developmental stage, instructional context, and expressive appraisal (Sundberg, 1987; Leman, 2007; Bremmer and Nijs, 2020; Ouyang, 2025). AI should therefore support human evaluative judgment, pedagogical dialogue, reflective uptake, and learners’ capacity for self-regulation rather than function as an autonomous evaluator of singing quality (de Bruin, 2024; Peng et al., 2026).

Several challenges follow from this view of AI-generated evidence. Inaccurate or poorly contextualized AI feedback may mislead learners if it is treated as authoritative correction rather than as evidence requiring pedagogical interpretation. Pitch tracking, vibrato detection, stability estimates, and effort-related cues may be affected by microphone distance, acoustics, accompaniment, fatigue, genre, and task. Over-reliance on AI feedback may also weaken learners’ internal monitoring when external indicators replace rather than support self-regulated practice. Algorithmic bias may arise not only from limited training data, but also from the evaluative assumptions embedded in what a system rewards or penalizes. A system optimized for smoothness, pitch regularity, or acoustic stability may mark stylistically legitimate breathiness, roughness, sliding pitch, straight tone, or genre-specific vibrato as error. In this way, AI feedback may push learners toward algorithmic conformity rather than stylistically appropriate vocal development. Transparency matters because teachers and learners need to know whether feedback is based on acoustic measurement, model-based inference, rule-based thresholds, language-model interpretation, or a combination of these processes. Data privacy is especially important because voice recordings, practice traces, and physiological or effort-related signals may reveal identity, emotional state, vocal condition, ability, and training history. Assessment context also matters: room acoustics, microphone distance, device quality, accompaniment, fatigue, task setting, genre, and learner level may influence the evidence AI systems generate. These issues reinforce the need for responsible, human-centered interpretation (UNESCO, 2023; OECD, 2023; Garcia Ramos and Wilson-Kennedy, 2024; Varsik and Vosberg, 2024).

The framework should also be adapted across vocal genres and learning contexts. Classical, popular, choral, musical theatre, and traditional singing may involve different vocal-aesthetic ideals, stylistic norms, technical priorities, and feedback vocabulary. For example, vibrato, straight tone, breathiness, voice quality, resonance, projection, and register balance may carry different pedagogical meanings across genres. A relatively straight tone may be desirable in some choral contexts, while vibrato may be central to other solo traditions. Breathiness or roughness may be stylistically meaningful in some popular or rock singing contexts, but less appropriate in other pedagogical settings. Similarly, falsetto, mixed voice, belting, and other register-related or voice-quality strategies may be evaluated differently in classical, musical theatre, and contemporary commercial styles. Learning contexts also matter. Conservatory training, school music education, adult or continuing education, professional coaching, amateur singing, and online or self-directed learning differ in learner readiness, teacher availability, assessment purpose, and the degree of mediation required. The framework should therefore be applied as a context-sensitive guide rather than as a universal scoring template.

Although this Perspective is grounded in vocal pedagogy, its broader relevance lies in a shared learning structure rather than direct equivalence across domains. Other performance-based learning contexts differ in technique and criteria, but they also rely on embodied skill, expert feedback, self-monitoring, context-sensitive judgment, and gradual transformation of practice. Work on embodied cognition and performative skill in music education and sport psychology supports this broader view of performance learning as interactive, social, and situated (Leman, 2007; McPherson et al., 2018; Bremmer and Nijs, 2020; Schiavio et al., 2019).

## 6 Conclusion

This Perspective began from a central problem in AI-assisted vocal pedagogy: more precise measurement does not automatically produce educational value. The framework proposed here links technical adaptation, human learning processes, and educational outcomes in order to explain how AI-generated vocal evidence can become useful for learning. Its main claim is that AI should not function as an autonomous evaluator of singing quality, but as a human-centered support for interpretation, reflective feedback use, teacher–student dialogue, and pedagogically responsible decision-making.

Future work should examine this framework in empirical settings. Comparative, design-based, longitudinal, and contextual studies can investigate how learners, teachers, experts, and AI systems interpret the same vocal evidence. They can also examine how AI-only, teacher-only, and teacher-mediated feedback shape trust, feedback uptake, learner agency, learning decisions, and pedagogical judgment. Such work should also examine how sustained and situated use of AI feedback in vocal teaching affects autonomy, dependence, expressive development, and learning transfer over time.

## Data Availability

The original contributions presented in the study are included in the article/supplementary material, further inquiries can be directed to the corresponding author.
